# Physical exercise prevents age-related heart dysfunction induced by high-salt intake and heart *salt*-specific overexpression in *Drosophila*

**DOI:** 10.18632/aging.203364

**Published:** 2021-08-12

**Authors:** Deng-Tai Wen, Lan Zheng, Kai Lu, Wen-Qi Hou

**Affiliations:** 1 Key Laboratory of Physical Fitness and Exercise Rehabilitation of Hunan Province, Hunan Normal University, Changsha 410012, Hunan Province, China; 2 Ludong University, Yantai 264025, Shandong Province, China

**Keywords:** heart aging, physical exercise, salt stress, oxidative stress

## Abstract

A long-term high-salt intake (HSI) seems to accelerate cardiac aging and age-related diseases, but the molecular mechanism is still not entirely clear. Exercise is an effective way to delay cardiac aging. However, it remains unclear whether long-term exercise (LTE) can protect heart from aging induced by high-salt stress. In this study, heart *CG2196(salt)* specific overexpression (HSSO) and RNAi (HSSR) was constructed by using the UAS/hand-Gal4 system in *Drosophila*. Flies were given exercise and a high-salt diet intervention from 1 to 5 weeks of age. Results showed that HSSR and LTE remarkably prevented heart from accelerated age-related defects caused by HSI and HSSO, and these defects included a marked increase in heart period, arrhythmia index, malondialdehyde (MDA) level, salt expression, and dTOR expression, and a marked decrease in fractional shortening, SOD activity level, dFOXO expression, PGC-1α expression, and the number of mitochondria and myofibrils. The combination of HSSR and LTE could better protect the aging heart from the damage of HSI. Therefore, current evidences suggested that LTE resisted HSI-induced heart presenility via blocking CG2196(salt)/TOR/oxidative stress and activating dFOXO/PGC-1α. LTE also reversed heart presenility induced by cardiac-salt overexpression via activating dFOXO/PGC-1α and blocking TOR/oxidative stress.

## INTRODUCTION

Aging is accompanied by a decline in heart function, which is associated with abnormal changes in the heart at the tissue, cellular, and molecular level, but the molecular basis for age-related heart function deterioration is multifaceted and not entirely clear [1]. In both mammals and fruit flies, heart aging is mainly characterized by cardiac contractile performance reduction, heart period prolongation, arrhythmia increase, myocardial hypertrophy, and myofibrils and mitochondria decreased [2, 3]. Besides, for molecular levels, heart aging inhibits FOXO and PGC-1α activity, it keeps TOR hyperactive, and it increases oxidative stress. The target of Rapamycin (TOR) is a nutrient sensor, and TOR mediates the increase in lifespan induced by caloric restriction (CR). Orchestrating metabolic homeostasis is also regulated by TOR pathway [4, 5]. Moreover, mitochondrial biogenesis and energy metabolism are regulated by PGC-1α in cardiac tissue, and a striking feature of age-related heart disease is a reduction in PGC-1α expression, and the activation of PGC-1α is also believed to be an important molecular mechanism of CR against aging [2, 6]. Next, FOXO can modulate the aging and longevity, and it is also involved in regulating cardiac aging, such as heart-specific *dFOXO*-overexpression decreases the stiffness, arrhythmia, pacing-induced heart failure, and diastolic interval, and it increases heart rate, myocardial relengthening rate, and cardiac output [7, 8]. Increasing evidence has shown that oxidative stress is an important mechanism leading to heart aging [9, 10]. Thus, heart aging is associated with cardiac structure and function degradation, PGC-1α and FOXO inhibition, TOR hyperactivation, and increased oxidative stress.

A long-term high-salt intake (HSI) seems to accelerate cardiac aging and age-related diseases. Firstly, in both mammals and *Drosophila*, it has been reported that excessive salt intake reduces the lifespan [11, 12]. Then, in mammals, a high-salt diet leads to the acceleration of cardiac interstitial fibrosis and perivascular fibrosis, and it eventually causes the deterioration of the cardiac function [13]. Moreover, cardiac hypertrophy can be induced by an unhealthy lifestyle such as a HSI. The response of cardiomyocytes to pathological stress can lead to cardiac hypertrophy, which is the result of ventricular wall thickening, and heart failure can be induced by chronic cardiac hypertrophy [14]. Finally, a long-term HSI is an unhealthy dietary mode, which can disturb the homeostasis of cardiomyocytes, cause mitochondrial dysfunction, and reduce the generation of ATP [15]. Therefore, a HSI can induce heart remodeling and heart failure, which seems to accelerate heart aging, and the mechanism responsible for HSI-induced heart aging is closely related to oxidative stress [16–18]. However, it is still unclear whether the mechanism of HSI-induced heart aging is related to the TOR, the FOXO, and the PGC-1α activity.

It has been reported that *CG2196(salt)* gene determines the salt tolerance of fruit flies, and it also seems to be linked to aging. The *salt* gene sequence location is 3R: 31751825…31754705. It is involved in the biological process described with: transmembrane transport; sodium ion transport. It is homologous to human SLC5A12 (solute carrier family 5 member 12) and SLC5A8 (solute carrier family 5 member 8) [19]. Overexpression of *salt* gene increased the salt stress in normal-diet flies, and it decreases their lifespan, which is similar to the results of high-salt intake in fruit flies. On the contrary, *salt* gene knockdown alleviates high-salt stress and increases the lifespan of high-salt-diet flies [12]. However, little is known about the function of the CG2196*(salt)* gene in the heart.

Exercise is an inducible form of physiologic stress, and it is considered an effective way to delay cardiac aging. In aging mammals, increasing evidence confirms that long-term moderate exercise training decreases abnormal cardiac remodeling, left ventricular dilation, myocardial fibrosis, mitochondrial dysfunction, and cardiac dysfunction, and it improves heart function and quality of life [20–22]. In *Drosophila*, increasing evidence shows that a long-term endurance exercise enhances cardiac function and delay heart age-related phenotypes, such as it can increase fraction shortening and the myocardial mitochondria in aged heart [10, 23, 24]. Besides, a long-term endurance exercise can prevent heart premature aging induced by a high fat diet in fruit flies [25]. The mechanism of exercise delay heart aging is also related to the TOR, the FOXO, and the PGC-1α activity [6, 10, 26, 27]. However, it remains unknown whether a long-term endurance exercise can efficiently prevent heart presenility induced by a long-term high-salt stress.

In this experiment, to explore whether a long-term endurance exercise can efficiently prevent heart presenility induced by a long-term high-salt stress, *w^1118^* flies were fed a high-salt diet and taken exercise from one week old to five weeks old, and then by using the UAS/hand-Gal4 system, cardiac *salt* gene was knocked down or over expressed in *Drosophila*. The cardiac *salt*, *dTOR*, *dFOXO*, and *PGC-1* gene expression level was tested by qRT-PCR. The systolic period, diastolic period, heart period, fractional shortening, diastolic diameter, systolic diameter, and arrhythmia index were measured by an M-mode trace. Finally, the heart SOD activity level and malondialdehyde(MDA) level were measured. Based on these indicators, we tried to understand the relationship between exercise, high-salt stress, oxidative stress, and cardiac aging.

## RESULTS

### High-salt intake(HSI) promoted age-related heart dysfunction, *dFOXO/PGC-1α* decline, and oxidative stress/ dTOR increase

In mammals, both HSI and aging lead to the myocyte hypertrophy, myocardial fibrosis, and mitochondrial dysfunction, which are more likely to end up with the deterioration of the cardiac function and heart failure [3, 13]. However, it is still unclear whether HSI can promote cardiac senescence and cardiac *CG2196(salt)* gene expression.

**Figure 1:**
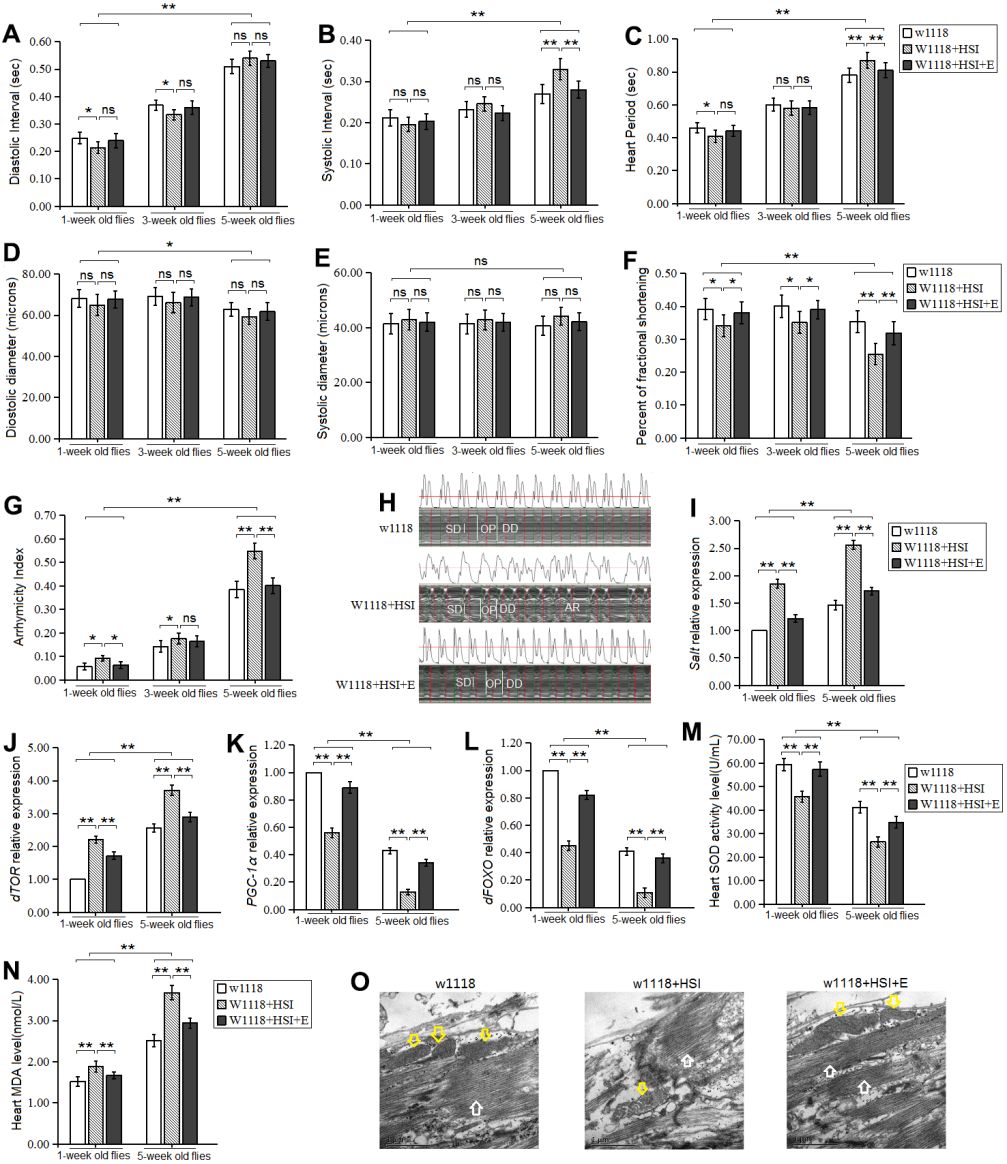
**Influence of HSI and LTE on heart.** (**A**) Heart diastolic period. (**B**) Heart systolic period. (**C**) Heart period. (**D**) Diastolic diameter. (**E**) Systolic diameter. (**F**) Fractional shortening. (**G**) Arrhythmia index. (**H**) Microscopic image of cardiac function from M-mode trace in 5-week-old Drosophila. It can be observed that HSI could increase heart period and arrhythmia, and it could reduce fractional shortening. LTE reduced heart period and arrhythmia, and it could increase fractional shortening. (**I**) Cardiac salt expression level. (**J**) Cardiac dTOR expression. (**K**) Cardiac PGC-1α expression. (**L**) Cardiac dFOXO expression. (**M**) Cardiac SOD activity level. (**N**) Cardiac MDA level. (**O**) Ultrastructure images of myocardium in 5-week old flies and the white arrows refer to the myofibril in the images, and the yellow arrows refer to mitochondria. Independent-sample t-tests were used to assess differences in 1-week old flies and 5-week old flies in flies. Using a one-way analysis of variance (ANOVA) followed by an LSD test among the group w1118, w1118+HSI, and w1118+HSI+E. Data are represented as means ± SEM. *P<0.05; **P <0.01. Sample size was 30 hearts for each group. For RT-PCR and ELISA, sample size was 80 hearts for each group, and measurements were taken 3 times.

In here, our results displayed that in one-week old and five-week old flies, a HSI dramatically reduced heart diastolic interval, heart period, and fractional shortening (*P<0.05, P<0.05, P<0.01*) (Figure 1A, 1C, 1F), and it dramatically increased arrhythmia index (*P<0.05*) (Figure 1G), but it did not dramatically change the heart systolic interval, diastolic diameter, and systolic diameter (*P>0.05*) (Figure 1B, 1D, 1E). In five-week old flies, a HSI dramatically reduced fractional shortening (*P<0.05*) (Figure 1F), and it dramatically increased heart systolic interval, heart period, arrhythmia index (*P<0.05 or P<0.01*) (Figure 1B, 1C, 1G, 1H), but it did not dramatically change the heart diastolic interval and diastolic diameter effectively (*P>0.05*) (Figure 1A, 1D).

Besides, in one-week old flies and five-week old flies, a HSI dramatically up regulated the heart *CG2196(salt)* gene expression (*P<0.01*) (Figure 1I). Moreover, a HSI dramatically up regulated the heart *dTOR* gene expression in both one-week old flies and five-week old flies (*P<0.01, P<0.01*) (Figure 1J), and it down regulated the heart *dFOXO* and *PGC-1* gene expression in both one-week old flies and five-week old flies (*P<0.01, P<0.01*) (Figure 1K, 1L). Since the myofibrils and mitochondria of cardiomyocytes are critical to the contractile function of the heart, the ultrastructure of cardiomyocytes was observed by electron microscopy in 5-week old flies. The images showed that a HSI reduced the number of myofibrils and mitochondria, and it destroyed the arrangement of myofibrils (Figure 1O).

In both *w^1118^* and *w^1118^*+HSI flies, aging dramatically increased diastolic interval, systolic interval, heart period, and arrhythmia index(*P<0.01*) (Figure 1A–1C, 1G), and it dramatically decreased diastolic diameter and fractional shortening (*P<0.05 or P<0.01*) (Figure 1D, 1F), but aging did not dramatically change the heart systolic diameter and (*P>0.05*) (Figure 1A). Aging dramatically up regulated the heart *CG2196 (salt)* gene expression, *dTOR* gene expression, and MDA level (*P<0.01*) (Figure 1I, 1J, 1N). Aging down regulated the heart SOD level, *dFOXO* expression, and *PGC-1α* gene expression (*P<0.01, P<0.01*) (Figure 1K–1M).

Thus, the results suggested that both long-term HSI and aging contributed to age-related accelerated decline of cardiac contractility and age-related accelerated increase of arrhythmias, and the mechanism may be related to up-regulation of heart *salt* gene expression and oxidative stress/dTOR pathway, and down-regulation of heart dFOXO/PGC-1α pathway. However, the effect of the heart salt gene on heart aging was unknown.

### Heart *salt* specific overexpression(HSSO) promoted age-related heart dysfunction, *dFOXO/PGC-1α* decline, and oxidative stress/ dTOR increase

HSI up regulates the *CG2196(salt)* gene expression, and it reduces the lifespan of flies. *CG2196(salt)* gene overexpression also reduces the survival of flies, and it seems to accelerate aging of flies [12]. However, it remains unknown the relationship between heart *salt* gene and heart aging.

**Figure 2:**
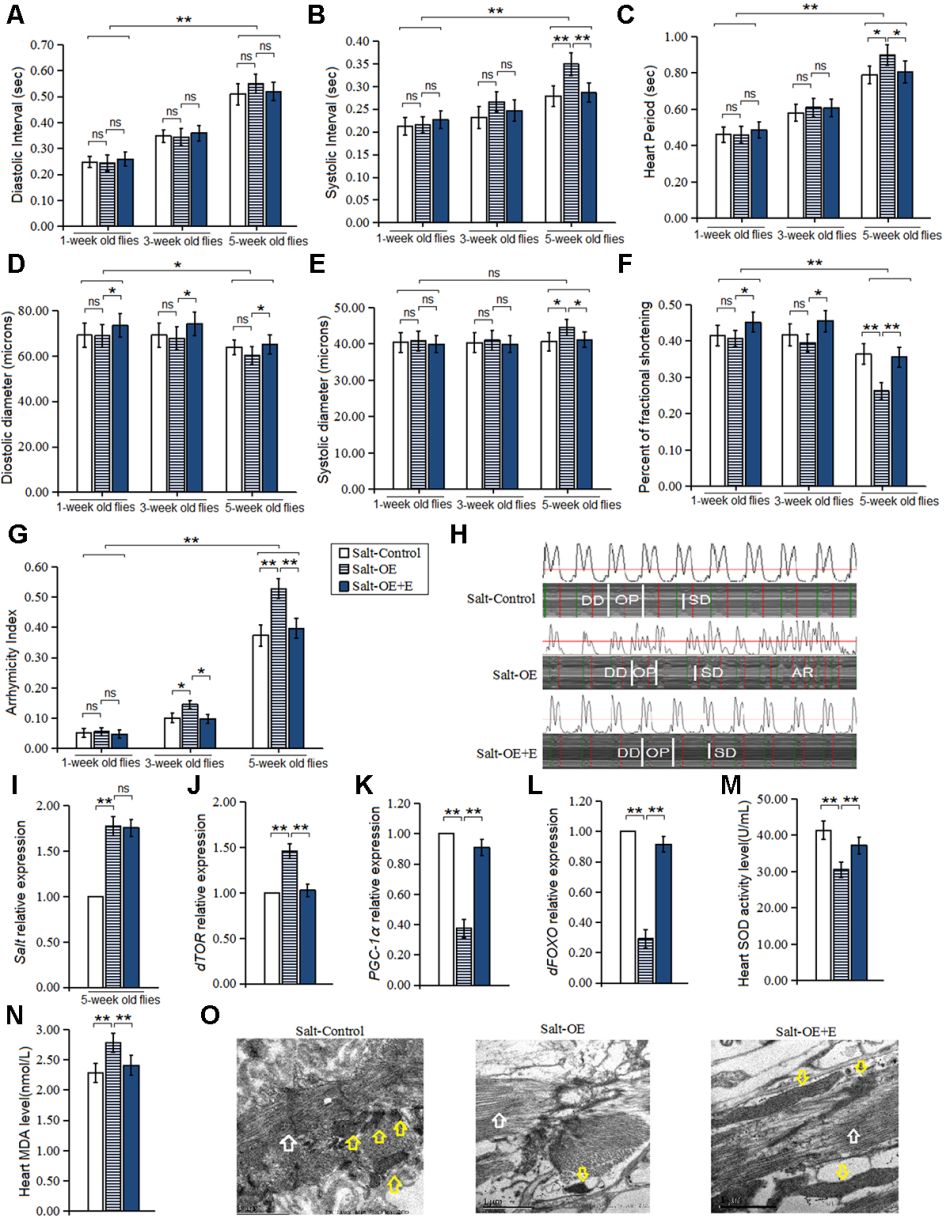
**Influence of salt overexpression and LTE on heart.** (**A**) Heart diastolic period. (**B**) Heart systolic period. (**C**) Heart period. (**D**) Diastolic diameter. (**E**) Systolic diameter. (**F**) Fractional shortening. (**G**) Arrhythmia index. (**H**) Microscopic image of cardiac function from M-mode trace in 5-week-old Drosophila. It can be observed that heart salt gene overexpression could increase heart period and arrhythmia, and it could reduce fractional shortening. LTE could reduce heart period and arrhythmia, and it could increase fractional shortening in heart salt gene overexpression flies. (**I**) Cardiac salt expression level. (**J**) Cardiac dTOR expression. (**K**) Cardiac PGC-1α expression. (**L**) Cardiac dFOXO expression. (**M**) Cardiac SOD activity level. (**N**) Cardiac MDA level. (**O**) Ultrastructure images of myocardium in 5-week old flies and the white arrows refer to the myofibril in the images, and the yellow arrows refer to mitochondria. Independent-sample t-tests were used to assess differences in 1-week old flies and 5-week old flies in flies. Using a one-way analysis of variance (ANOVA) followed by an LSD test among the group Salt-control, Salt-OE, and Salt-OE+E. Data are represented as means ± SEM. *P<0.05; **P <0.01. Sample size was 30 hearts for each group. For RT-PCR and ELISA, sample size was 80 hearts for each group, and measurements were taken 3 times.

In this experiment, the HSSO was produced by using UAS/hand-gal4 system, and the HSSO flies were raised up to five weeks of age. Our results displayed that the heart *salt* gene expression of *salt*-OE flies was higher than that of *salt*-control group flies (*P<0.01*) (Figure 2I), which indicated that HSSO was built successfully. Besides, HSSO dramatically increased the systolic interval, heart period, and systolic diameter in five-week old flies (*P<0.05 or P<0.01*) (Figure 2B, 2C, 2E). In three-week old flies and five-week old flies, HSSO dramatically decreased the fractional shortening in 5-week old flies (*P<0.01*) (Figure 2F), and it dramatically increased arrhythmia index (*P<0.05 or P<0.01*) (Figure 2G, 2H).

In addition, our results showed that HSSO dramatically up regulated heart *dTOR* expression (*P<0.01*) (Figure 2J), and it dramatically increased MDA level in five-week old flies (*P<0.01*) (Figure 2N). However, HSSO dramatically down regulated heart *dFOXO* and *PGC-1α* gene expression (*P<0.01, P<0.01*) (Figure 2K, 2L), and it decreased SOD activity level (*P<0.01, P<0.01*) (Figure 2M). Finally, the electron microscopy images showed that HSSO decreased the number of myofibrils and mitochondria, and it disrupted the arrangement of myofibrils (Figure 2O).

In both *Salt-*Control and *Salt-*OE flies, aging dramatically increased diastolic interval, systolic interval, heart period, diastolic diameter, and arrhythmia index(*P<0.01*) (Figure 2A–2D, 2G), and it dramatically decreased fractional shortening (*P<0.05 or P<0.01*) (Figure 2F), but aging did not dramatically change the heart systolic diameter (*P>0.05*) (Figure 2E).

Therefore, these results suggested that HSSO also promoted age-related impairment of heart contractility and an increase in age-related arrhythmias, and the mechanism of that may be related to up-regulation of heart oxidative stress/dTOR pathway and down-regulation of heart dFOXO/PGC-1α pathway.

**Figure 3:**
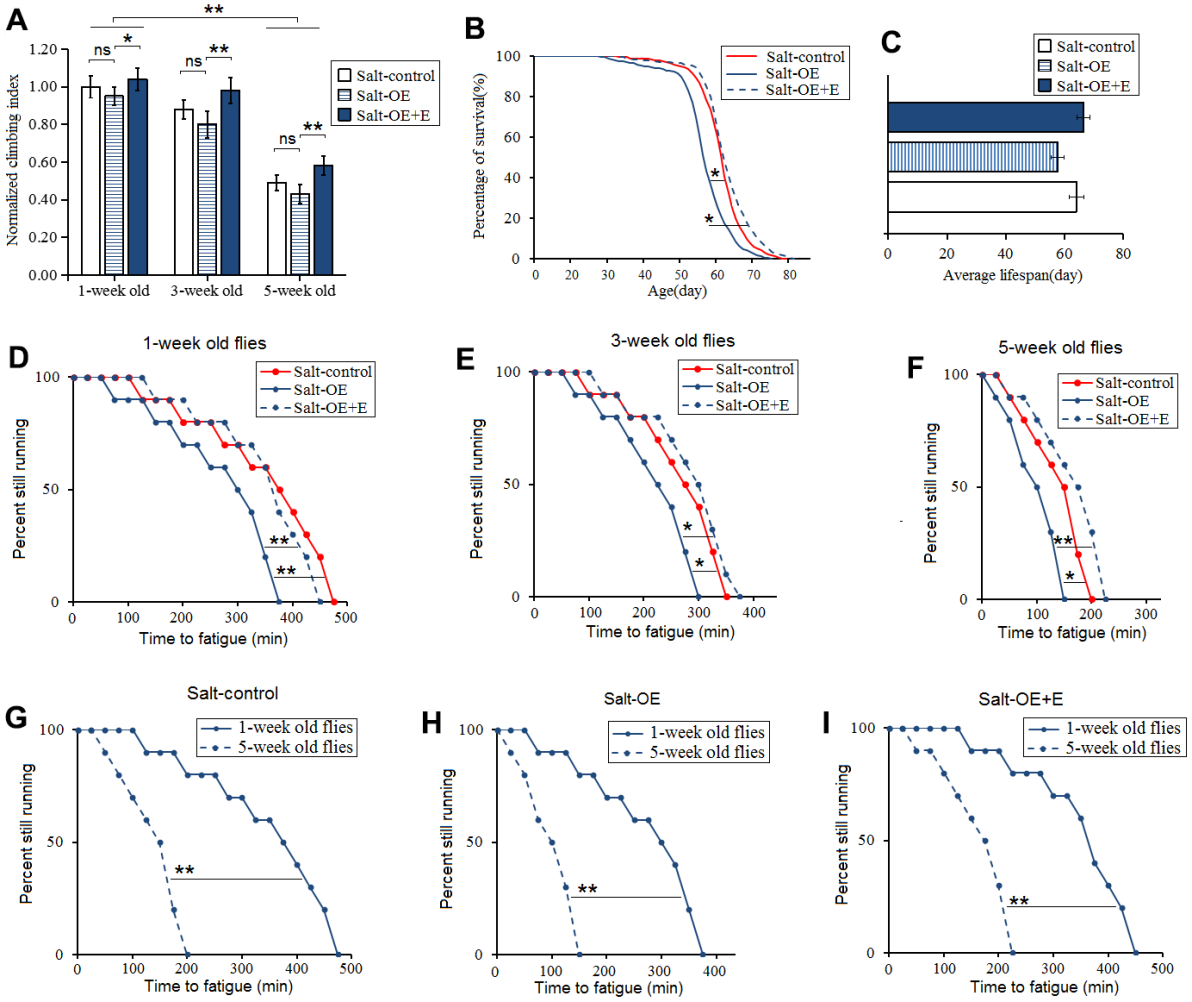
**Influence of heart salt gene overexpression and LTE on the climbing ability and lifespan in Drosophila.** (**A**) The climbing index in 1-week old, 3-week old, and 5-week old flies. (**B**) Fly population survival (%)curve. The leftmost curve represents the salt-OE group, of which flies had the shortest lifespan. (**C**) The average lifespan. (**D**) The climbing endurance in 1-week old flies. (**E**) The climbing endurance in 3-week old flies. (**F**) The climbing endurance in 5-week old flies. (**G**) The climbing endurance of Salt-control. (**H**) The climbing endurance of Salt-OE. (**I**) The climbing endurance of Salt-OE+E. The leftmost curve represents the salt-OE group, of which flies had the shortest lifespan. For climbing ability measurement, the sample size was about 100 flies for each group. For climbing index, using a one-way analysis of variance (ANOVA) followed by an LSD test among these groups. For lifespan, the sample size was 200-220 flies for each group. P-values for lifespan curves and climbing endurance curves were calculated by the log-rank test. Data are represented as means ± SEM. *P<0.05; **P <0.01.

Moreover, to explore the relationship between HSSO and flies body aging, the climbing ability and lifespan were also measured. Our results showed that HSSO did not dramatically change the climbing index in aging flies (*P>0.05*) (Figure 3A), but it dramatically reduced the climbing endurance in 1-week old, 3-week old, and 5-week old flies (*P<0.05 or P<0.01*) (Figure 3D, 3E, 3F). Moreover, HSSO dramatically reduced the lifespan of flies (*P<0.05*) (Figure 3B, 3C). Aging dramatically reduced the climbing index and climbing endurance (*P<0.01*) (Figure 3A, 3G, 3H, 3I). These results indicated that HSSO promoted aging-related decline in exercise capacity and hastened aging-related death.

### Long-term exercise (LTE) protected the heart from age-related dysfunction caused by HIS

Numerous studies have shown that LTE can enhance the heart's ability to contract, reduce cardiac dysfunction, and delay cardiac aging [21, 28–30].

Increasing evidence confirms that LTE can improve the heart defects induced by high sugar diet, high fat diet (HFD), or high-salt diet [25, 31–33]. However, it remains unknown whether LTE can prevent heart presenility induced b**y** HSI when LTE and HSI are present throughout lifetime of flies. The relationship between LTE and the heart *salt* gene is also unclear.

In this experiment, our results showed that in one-week old HSI flies, LTE dramatically increased the heart fractional shortening (*P<0.05*) (Figure 1F), and it dramatically decreased arrhythmia index (*P<0.05*) (Figure 1G), but LTE did not dramatically change the heart systolic diameter, diastolic diameter, systolic interval, diastolic interval, and heart period (*P>0.05*) (Figure 1A–1E). In 3-week old HSI flies, LTE dramatically increased the heart fractional shortening (*P<0.05*) (Figure 1F), but LTE did not dramatically change the heart systolic diameter, diastolic diameter, systolic interval, diastolic interval, heart period, and arrhythmia index (*P>0.05*) (Figure 1A–1E, 1G). In five-week old HSI flies, LTE dramatically decreased the heart systolic interval, heart period, and arrhythmia index (*P<0.01*) (Figure 1B, 1C, 1G), and it dramatically increased fractional shortening (*P<0.01*) (Figure 1F, 1H), but it did not effectively change the heart systolic diameter, diastolic diameter, and diastolic interval (*P>0.05*) (Figure 1A, 1D, 1E).

Besides, our results showed that in 1-week old HSI flies, LTE dramatically down regulated the heart *CG2196(salt)* gene expression (*P<0.01*) (Figure 1I) and *dTOR* gene expression(*P<0.05*) (Figure 1J), and it dramatically decreased MDA level (*P<0.05*) (Figure 1N). LTE dramatically up regulated the heart *dFOXO* and *PGC-1α* gene expression (*P<0.01, P<0.01*) (Figure 1K, 1L), and LTE increased SOD activity level(*P<0.01*) (Figure 1M). In 5-week old HSI flies, LTE also dramatically down regulated the heart *CG2196(salt)* gene expression (*P<0.01*) (Figure 1I) and *dTOR* gene expression(*P<0.01*) (Figure 1J), and it dramatically decreased MDA level (*P<0.05*) (Figure 1N), but LTE up regulated the heart *dFOXO* and *PGC-1α* gene expression (*P<0.01, P<0.01*) (Figure 1K, 1L), and it increased SOD activity level(*P<0.01*) (Figure 1M). The electron microscopy images showed that LTE increased the number of myofibrils and mitochondria, and it enhanced the arrangement of myofibrils (Figure 1O).

In w^1118^+HSI+E flies, aging dramatically increased diastolic interval, systolic interval, heart period, and arrhythmia index(*P<0.01*) (Figure 1A–1C, 1G), and it dramatically decreased diastolic diameter and fractional shortening (*P<0.05 or P<0.01*) (Figure 1D, 1F), but aging did not dramatically change the heart systolic diameter and (*P>0.05*) (Figure 1A). Aging dramatically up regulated the heart *CG2196 (salt)* gene expression, *dTOR* gene expression, and MDA level (*P<0.01*) (Figure 1I, 1J, 1N). Aging down regulated the heart *dFOXO* expression, *PGC-1α* gene expression, and SOD level (*P<0.01, P<0.01*) (Figure 1K–1M).

Thus, these results suggested that LTE prevented age-related accelerated decline of cardiac contractility and age-related accelerated increase of arrhythmias induced by a HSI, and the mechanism of that may be related to dow-regulation of heart *salt* gene expression and oxidative stress/dTOR pathway, and up-regulation of heart dFOXO/PGC-1α pathway.

### Long-term exercise (LTE) improved age-relate heart dysfunction induced by HSSO

To explore whether LTE could resist the damages induced by HSSO in flies, the HSSO flies were taken exercise from 1-week old to 5-week old. Previous studies have confirmed that exercise training can prevent heart defects, mobility decline, and lifespan reduction induced by *CG9940* gene or *dSir2* gene mutation [23–25]. Therefore, as a mild intervention, exercise training has potential therapeutic significance for genetic diseases.

In this experiment, our results showed that in one-week old HSSO flies, LTE dramatically increased the diastolic diameter and heart fractional shortening (*P<0.05*) (Figure 2D, 2F), but LTE did not dramatically change the heart systolic diameter, diastolic diameter, systolic interval, diastolic interval, heart period, and arrhythmia index (*P>0.05*) (Figure 2A–2C, 2E, 2G). In three-week old HSSO flies, the diastolic diameter and heart fractional shortening (*P<0.05*) (Figure 2D, 2F), and it dramatically decreased arrhythmia index (*P<0.05*) (Figure 2G), but LTE did not dramatically change the heart systolic diameter, systolic interval, diastolic interval, and heart period (*P>0.05*) (Figure 2A–2E, 2G). In 5-week old HSSO flies, LTE dramatically decreased the heart systolic interval, heart period, systolic diameter, and arrhythmia index (*P<0.01*) (Figure 2B, 2C, 2E, 2G, 2H), and it dramatically increased diastolic diameter and fractional shortening (*P<0.05*) (Figure 2D, 2F), but LTE did not effectively change the heart diastolic diameter (*P>0.05*) (Figure 2A).

In 5-week old HSSO flies, LTE also dramatically down regulated the heart *dTOR* gene expression (*P<0.01*) (Figure 2J), and it dramatically decreased MDA level (*P<0.05*) (Figure 2N), but LTE up regulated the heart *dFOXO* and *PGC-1α* gene expression (*P<0.01*) (Figure 2K, 2L), and it increased SOD activity level(*P<0.01*) (Figure 2M). The electron microscopy images showed that LTE increased the number of myofibrils and mitochondria, and it enhanced the arrangement of myofibrils (Figure 2O). LTE did not effectively change the heart salt expression level(*P>0.05*) (Figure 2I).

In Salt-OE+E flies, aging dramatically increased diastolic interval, systolic interval, heart period, and arrhythmia index (*P<0.01*) (Figure 2A–2C, 2G), and it dramatically decreased diastolic diameter and fractional shortening (*P<0.05 or P<0.01*) (Figure 2D, 2F), but aging did not dramatically change the heart systolic diameter and (*P>0.05*) (Figure 2A).

Therefore, LTE prevented age-related accelerated decline of cardiac contractility and age-related accelerated increase of arrhythmias induced by HSSO, and the mechanism of that may be related to down-regulation of oxidative stress/dTOR pathway and up-regulation of heart dFOXO/PGC-1α pathway.

Our results showed that LTE dramatically increased the climbing index, climbing endurance, and survival in HSSO flies (*P<0.05 or P<0.01*) (Figure 3A–3F), and aging dramatically reduced the climbing index and climbing endurance(*P<0.01*) (Figure 3A, 3D, 3F, 3F). These indicated that LTE resisted the body aging induced by HSSO.

### Heart *salt* specific RNAi(HSSR) prevented heart age-related abnormalities induced by HSI

**Figure 4:**
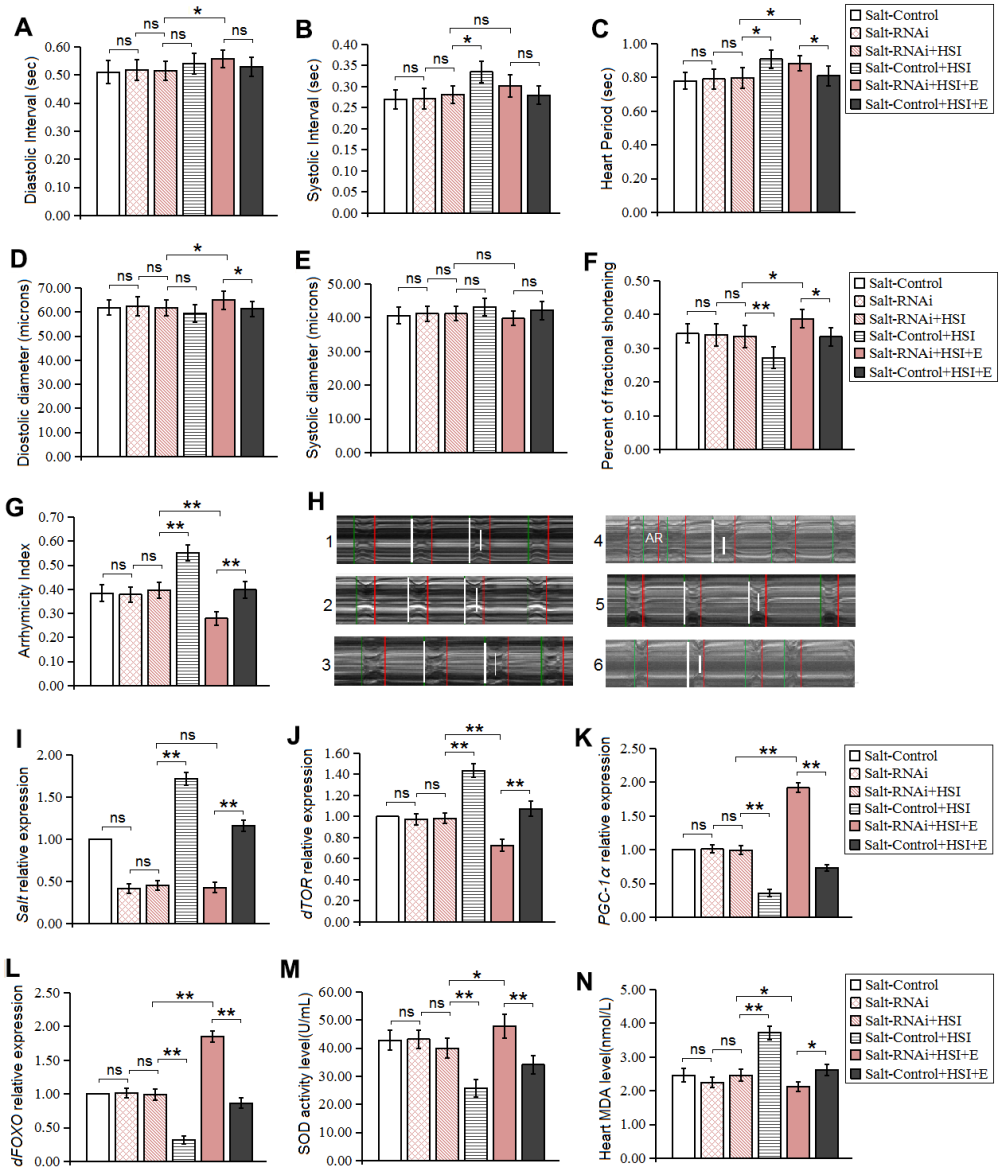
**Influence of salt RNAi, HSI, and LTE on heart.** (**A**) Heart diastolic period. (**B**) Heart systolic period. (**C**) Heart period. (**D**) Diastolic diameter. (**E**) Systolic diameter. (**F**) Fractional shortening. (**G**) Arrhythmia index. (**H**) Microscopic image of cardiac function from M-mode trace in 5-week-old Drosophila. It can be observed that heart salt gene overexpression could increase heart period and arrhythmia, and it could reduce fractional shortening. LTE could reduce heart period and arrhythmia, and it could increase fractional shortening in heart salt gene overexpression flies. (**I**) The heart salt expression level. (**J**) The heart dTOR expression. (**K**) Cardiac PGC-1α expression. (**L**) Cardiac dFOXO expression. (**M**) Cardiac SOD activity level. (**N**) Cardiac MDA level. Using a one-way analysis of variance (ANOVA) followed by an LSD test among these groups. Data are represented as means ± SEM. *P<0.05; **P <0.01. Sample size was 30 hearts for each group. For RT-PCR and ELISA, sample size was 80 hearts for each group, and measurements were taken 3 times.

It has been reported that *salt* gene knockdown in flies can prevent lifespan decrease from high salt diet, but it remains unknown whether knock-down of heart *salt* gene can resist heart presenility induced by HSI. In this experiment, our results displayed that heart *salt* expression of *salt*-RNAi flies was dramatically lower than that of *salt*-control flies (*P<0.01*) (Figure 4I), which indicated that HSSR was built successfully.

Our results showed that in five-week old flies, HSSR didn't dramatically change heart diastolic diameter, systolic diameter, fractional shortening, diastolic interval, systolic interval, heart period, arrhythmia index, SOD activity level, MDA level, *dTOR* gene, *dFOXO* gene, and *PGC-1α* gene expression (*P>0.05*) (Figure 4A–4H, 4J–4N). These indicated that HSSR did not dramatically delay the natural aging of the heart. Besides, in 5-week old HSSR flies, HSI didn't dramatically change heart diastolic diameter, systolic diameter, fractional shortening, diastolic interval, systolic interval, heart period, arrhythmia index, SOD activity level, MDA level, *dTOR* gene, *dFOXO* gene, and *PGC-1α* gene expression (*P>0.05*) (Figure 4A–4H, 4J–4N).

In five-week old HSI flies, HSSR dramatically decreased systolic interval, heart period, and arrhythmia index (*P<0.05 or P<0.01*) (Figure 4B, 4C, 4G, 4H), and it dramatically increased fractional shortening (*P<0.01*) (Figure 4F). Moreover, HSSR dramatically decreased salt expression, dTOR expression, and MDA level(*P<0.01*) (Figure 4I, 4J, 4N), and it dramatically increased up regulated the heart *dFOXO* gene expression, *PGC-1α* gene expression, and SOD activity level(*P<0.01*) (Figure 4K, 4L, 4M).

**Figure 5:**
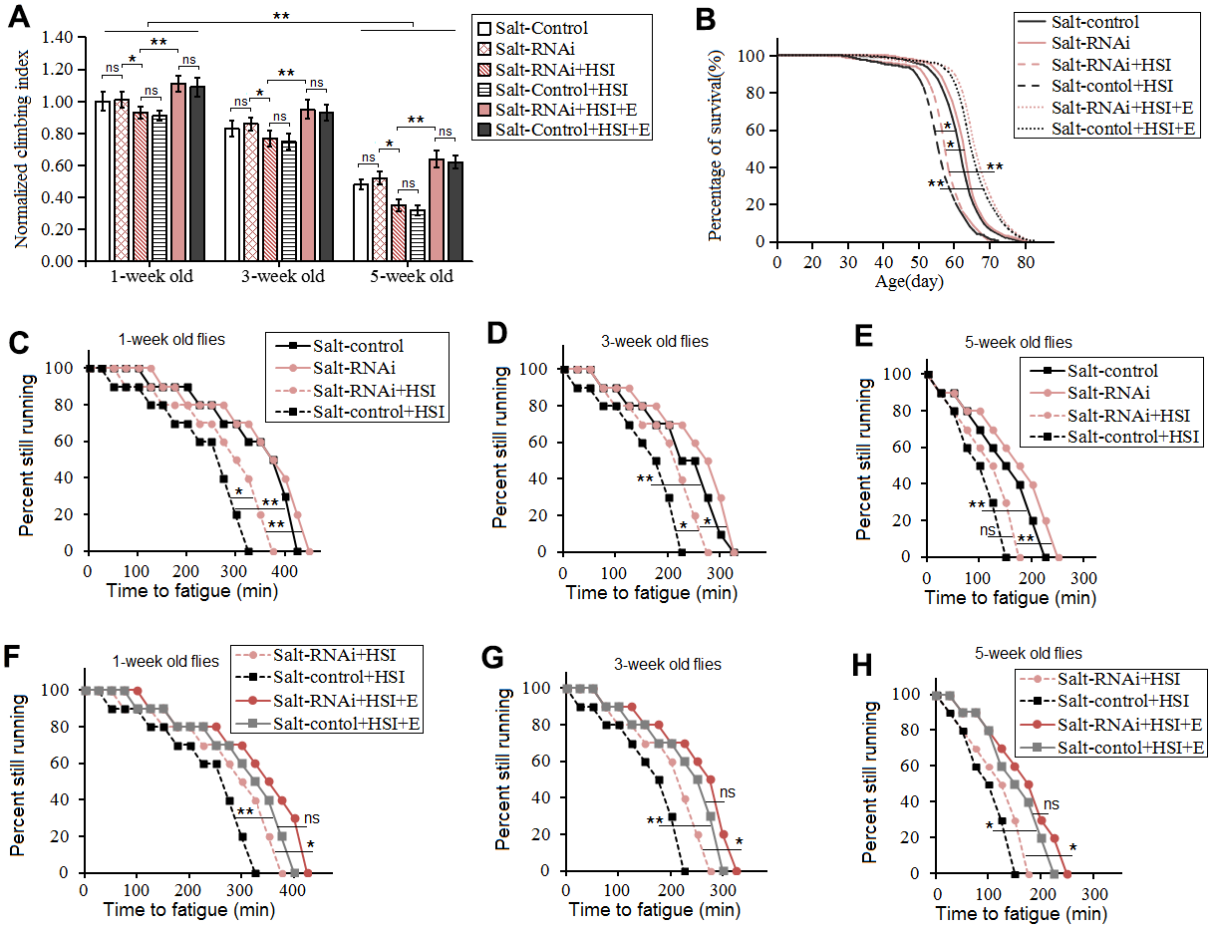
**Influence of heart salt RNAi, HSI, and LTE on the climbing ability and lifespan in Drosophila.** (**A**) The climbing index in 1-week old, 3-week old, and 5-week old flies. (**B**) Fly population survival (%) curve. The leftmost curve represents the Salt-control+HSI group, of which flies had the shortest lifespan. (**C**) Influence of heart salt RNAi and HSI on climbing endurance in 1-week old flies. (**D**) Influence of heart salt RNAi and HSI on climbing endurance in 3-week old flies. (**E**) Influence of heart salt RNAi and HSI on climbing endurance in 5-week old flies. (**F**) Influence of heart salt RNAi, exercise, and HSI on climbing endurance in 1-week old flies. (**G**) Influence of heart salt RNAi, exercise, and HSI on climbing endurance in 3-week old flies. (**H**) Influence of heart salt RNAi, exercise, and HSI on climbing endurance in 5-week old flies. For climbing ability measurement, the sample size was about 100 flies for each group. For climbing index, using a one-way analysis of variance (ANOVA) followed by an LSD test among different groups. For lifespan, the sample size was 200-220 flies for each group. P-values for lifespan curves and climbing endurance curves were calculated by the log-rank test. Data are represented as means ± SEM. *P<0.05; **P <0.01.

HSSR didn't dramatically change climbing index, climbing endurance, survival, and average lifespan in aging flies (*P>0.05*) (Figure 4A–4H). HSI dramatically reduced the climbing index, climbing endurance, survival, and average lifespan in 5-week old HSSR flies (*P<0.05 or P<0.01*) (Figure 5A–5H).

These indicated that HSSR did not delay the body aging induced by HSSO, but HSSO did resist heart presenility induced by HSI.

### Long-term exercise(LTE) delayed heart aging and body aging induced by HSI in HSSR *Drosophila*

To explore whether LTE combined with HSSR did better combat the harm induced by HSI, the HSSR flies were taken exercise training and fed a HSI from young to old.

Our results showed that LTE dramatically reduced diastolic interval, heart period heart, diastolic diameter, and fractional shortening in 5-week old HSSR-HSI flies (*P<0.05 or P<0.01*) (Figure 4A, 4C, 4D, 4F), and it dramatically decreased arrhythmia index (*P<0.01*) (Figure 4G, 4H), but it did not effectively change the heart systolic interval and systolic diameter (*P>0.05*) (Figure 4B, 4E). Next, in 5-week old HSSR-HSI flies, LTE dramatically down regulated the heart *dTOR* gene expression and decreased MDA level (*P<0.01*) (Figure 4J, 4N), and it increased SOD activity level and up regulated the heart *dFOXO* and *PGC-1α* gene expression (*P<0.01*) (Figure 4K, 4L, 4M), but it did not effectively change the heart *salt* gene expression (*P>0.05*) (Figure 4I).

In 5-week old HSI+E flies, HSSR dramatically increased heart period, diastolic diameter, and fractional shortening(*P<0.05*) (Figure 4C, 4D, 4F), but it dramatically decreased arrhythmia index (*P<0.01*) (Figure 4G), and HSSR didn't dramatically change diastolic interval, systolic interval, systolic diameter (*P>0.05*) (Figure 4A, 4B, 4E). Besides, in 5-week old HSI+E flies, HSSR dramatically decreased *salt* expression, dTOR expression, and MDA level(*P<0.01*) (Figure 4I, 4J, 4N), and it dramatically increased up regulated the heart *dFOXO* expression, *PGC-1α* gene expression, and SOD activity level(*P<0.01*) (Figure 4K, 4L, 4M).

Finally, In 5-week old HSI+E flies, LTE dramatically increased the climbing index, climbing endurance, and lifespan (*P<0.05 or P<0.01*) (Figure 5A–5H). In 5-week old HSI+E flies, HSSR did not dramatically change the climbing index, climbing endurance, and lifespan (*P<0.05 or P<0.01*) (Figure 5A–5H).

Therefore, these results indicated that LTE combined with HSSR further enhanced heart contractility and reduced arrhythmia in HSI flies, and the mechanism was related to down-regulation of heart *dTOR* expression and oxidative stress decrease, and up-regulation of heart *dFOXO* and *PGC-1α* gene expression. Besides, LTE could also resist whole body aging induced by a HSI in HSSR flies.

## DISCUSSION

In mammals, cardiac aging is majorly characterized by increased myocardial hypertrophy, fibrosis, contractility debility, and oxidative stress, and a HSI seems to speed up the aging of the heart. For example, the interventricular septum thickness, cardiomyocyte size, diastolic dysfunction, and preserved ejection fraction can be dramatically increased by feeding a high-salt diet, and this accelerates cardiac interstitial and perivascular fibrosis [34–37]. Besides, reactive oxygen species (ROS) production can be induced by high salinity, and this leads to oxidative damage [16, 17]. Oxidative stress-induced periostin is involved in myocardial fibrosis and hypertrophy [38, 39]. Moreover, mitochondria and oxidative stress are believed to be related to cardiac aging and the development of heart disease such as cardiac hypertrophy, diabetic cardiomyopathy, and heart failure, and the decrease of cardiac mitochondrial function and the accumulation of macromolecular oxidative damage may be the cause of the decline of cardiac function with age [40, 41]. Next, a large number of studies have confirmed that FOXO (Forkhead Box O) transcription factor is an important determinant of oxidative stress and aging [42–44]. However, oxidative stress also regulates the activities of FOXO proteins, and this induces the phosphorylation, translocation to the nucleus, and acetylation-deacetylation of FOXO [45]. What’s more, as a transcriptional coactivator of many genes, PGC-1α is involved in energy metabolism management and mitochondrial biogenesis, and the expression of PGC-1α is closely related to organismal aging, cellular senescence, and many age-related diseases [46]. Finally, TOR is involved in regulating cardiac development and cardiac function. Rapamycin can inhibit the TOR activity, which improves pathological cardiac hypertrophy and age-related cardiac functional decline, and decreases the activity of cardiac proteasome [47]. For example, when the activity of TOR is inhibited by rapamycin, the cardiac preserved ejection fraction and capillary structure will be enhanced, and cardiac left ventricular hypertrophy and fibrosis will be relieved [48]. In addition, rapamycin is an inhibitor of mammalian target of rapamycin (mTOR) involved in the regulation of stress [49]. Therefore, these evidences suggest that HSI may contribute to heart aging by increasing oxidative stress, activating mTOR activation, and inhibiting FOXO/PGC-1α activation.

Since the *Drosophila* has a suite of molecular and genetic tools, and these are highly conserved traits of cardiac senescence, the fruit fly has become a very classic model organism for studying cardiac aging in a short timeframe [1]. In flies, cardiac aging is also characterized by increased contractility debility, arrhythmia, and oxidative stress and so on [50–52]. So, the *Drosophila* has been used as an ideal model for studying the molecular mechanisms of HSI-induced cardiac aging. In this experiment, it is the first time to explore the relationship between high salt stress and the heart aging in flies. Our results suggested that both a HSI and heart *CG2196(salt)* specific overexpression(HSSO) decreased the heart fractional shortening, and it increased heart period and arrhythmia index. Besides, both HSI and HSSO up regulated heart *salt* and *dTOR* gene expression, increased MDA level, but it down regulated heart *dFOXO* and *PGC-1α* gene expression and reduced SOD activity level in aging flies. What’s more, we observed that both HSI and HSSO reduced the myofibrils and mitochondria in cardiomyocytes. However, we also found heart *CG2196(salt)* specific RNAi(HSSR) can effectively prevent heart dysfunction and premature aging induced by a HSI, but HSSR could not protected the climbing ability and lifespan from HSI-induced damages in flies.

In flies, *dTOR, dFoxo,* and *PGC-1α/srl* gene have been shown to regulate the progression of age-related decline in cardiac function [8, 10, 27, 53]. For instance, a HFD can induce heart conduction blocks and severe structural pathologies, and severe structural pathologies in flies, and reducing insulin-TOR activity by heart-specific overexpression of FOXO can effectively reduce cardiac lipid accumulation and dysfunction induced by HFD in fruit flies [54]. The lipid stores and glucose levels can be decreased by inhibiting the function of *Drosophila* TOR, and this is closely relate to the blocking of insulin resistance and metabolic syndrome phenotypes, and this is also closely relate to increased activity of the insulin responsive transcription factor, dFOXO. The age-dependent decline in heart function can be protected by reducing TOR activity, and longevity can be increased by reducing TOR activity [55]. Besides, decrease of PGC-1/srl function can lead to lipid accumulation and cardiac dysfunction, which is similar to heart defects induced by high fat diet. On the contrary, overexpression of PGC-1/srl protects against HFD-induced heart defects. TOR function as an upstream regulator of PGC-1/srl can be enhanced by HFD [56, 57]. In high-salt-diet flies, the climbing capacity, lifespan, and antioxidant capacity can be dramatically enhanced by overexpression of the systemic *dFOXO* gene, but overexpression of the systemic *dFOXO* gene can’t induce changes in the expression of *salt* gene [18]. While, oxidative stress regulates the activities of FOXO proteins, and this induces the phosphorylation, translocation to the nucleus, and acetylation-deacetylation of FOXO [45]. Therefore, these evidences suggested that both HFD and HSI contributed heart to presenility in aging flies. Up-regulation of *CG2196(salt)*/*dTOR* and down-regulation of dFOXO/*PGC-1/srl* were two important pathways of HSI-induced heart presenility. Importantly, as a key gene regulating cardiac salt tolerance, the expression of *CG2196(salt)* played a decisive role in the regulation of cardiac presenility induced by a HSI.

In mammals, exercise training (ET) can improve heart dysfunction induced by high salt stress. For example, swimming training leads to the improvement of cardiac contractility, relaxation and systolic capacity, and more pronounced effects of exercise in alleviating oxidative stress are observed in high-salt rats [58]. Besides, a HSI increases heart wall thicknesses and LV volumes, it decreases the deformation parameters, and it contributes to the development of insulin resistance, and it eventually leads to heart failure and cardiac hypertrophy. However, physical exercise enhances cardiac function, and it decreases the extent of interstitial fibrosis and insulin levels [59]. Moreover, high-intensity interval training improves the preserved left ventricular ejection fraction induced by high-salt stress, and the mechanism is that High-intensity interval training reverses the endothelial dysfunction such as nicotinamide adenine dinucleotide phosphate-oxidase, endothelial nitric oxide synthase, and advances glycation end product induced by high-salt stress [60]. Finally, ET can promote cardiac remodeling to some extent, and it decreases HF in hypertensive rats. ET may induce left ventricular concentricity attenuation and restoration of coronary angiogenesis through activation of phosphatidylinositol 3-kinase(p110alpha)-Akt-mTOR signaling [61]. The myocardial oxidative stress injury and apoptosis can be decreased by endurance training, and its molecular mechanism is linked to the activation of SIRT1 signaling pathway, up-regulation the myocardial expression of SIRT1 and the deacetylation of FOXO1 [62]. ET can up-regulate the expression levels of SIRT1 and PGC-1α proteins, which contributes to energy homeostasis and suppression of age-related inflammatory cytokines [63]. ET protects the heart by reducing oxidative stress and cardiac fibrosis and by improving the mitochondrial integrity and biogenesis in post-MI myocardium, and this is associated with the activation of SIRT1/PGC-1α/PI3K/Akt pathway [64]. Therefore, these evidences suggested that exercise training might resist heart presenility induced by a HSI via reducing oxidative stress and activating *FOXO* and *PGC-1α* in mammals.

In this experiment, we found that LTE could improve heart contractility reduction and arrhythmia increase induced by a HSI, and the mechanism was related to down-regulation of heart *salt* and *dTOR* gene expression, and up-regulation of heart *dFOXO* and *PGC-1* gene expression. LTE could resist heart presenility induced by a HSI. Besides, LTE could improve heart contractility reduction and arrhythmia increase induced by HSSO, and the mechanism was related to down-regulation of heart *dTOR* gene expression, and up-regulation of heart *dFOXO* and *PGC-1α* gene expression. LTE could also delay the aging of whole body in HSSO flies. Moreover, LTE combined with HSSR could further enhance heart contractility and reduce arrhythmia in HSI flies, and the mechanism was related to down-regulation of heart *dTOR* gene expression, and up-regulation of heart *dFOXO* and *PGC-1* gene expression. LTE could also resist whole body aging induced by a HSI in HSSR flies.

In flies, LTE can delay heart aging and resist heart presenility induced by a HFD or genetic defects. For example, LTE resists HFD-induced or cardiac-dSir2 knockdown-induced heart presenility in flies, and the mechanism is associated with up-regulation of heart the NAD^+^/dSIR2/PGC-1α pathway [27]. Next, HFD promotes age-related climb ability decline, cardiac dysfunction, mortality, and dSir2 expression decline, but lifelong endurance exercise can prevent that from happening and slow down the rapid aging induced by HFD in *Drosophila* [25]. Besides, overexpression of cardiac *dSir2* or low expression of cardiac *dSir2* reduces or increases age-related cardiac lipid accumulation, oxidative stress, diastolic dysfunction, and contractility debility, and the mechanism is associated with activation of cardiac *dSir2*/Foxo/SOD and dSir2/Foxo/bmm pathways [10]. Finally, inhibiting insulin-TOR activity by over-expressing FOXO efficiently alleviates cardiac dysfunction and cardiac lipid accumulation induced by a HFD. Therefore, these evidences suggested that LTE resisted heart presenility induced by a HSI, and the mechanism of that is it reduced oxidative stress, inhibited *CG2196(salt)/dTOR* activity, and activated dFOXO/PGC-1α. While, for heart presenility induced by cardiac salt overexpression, LTE could reverse it by inhibiting *dTOR* activity and activating dFOXO/PGC-1α, but LTE could reverse it not through changing cardiac *salt* gene expression.

## CONCLUSIONS

**Figure 6:**
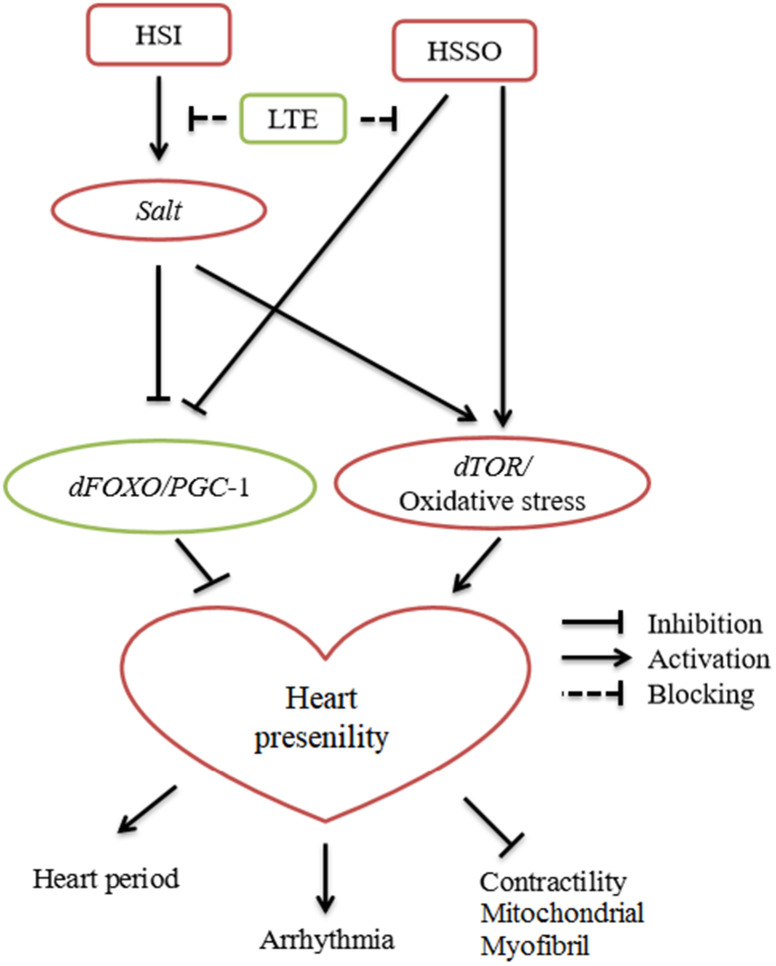
Current evidences suggested the relationship between high-salt intake (HSI), exercise training (ET), heart salt specific overexpression (HSSO), and heart presenility.

Current evidences suggested that the *CG2196(salt)/TOR/*oxidative stress and *dFOXO/PGC-1α* may play a important role in the regulation of cardiac presenility induced by a HSI. LTE resisted HSI-induced heart presenility via blocking *CG2196(salt)/TOR/*oxidative stress and activating *dFOXO/PGC-1α*. LTE also reversed heart presenility induced by cardiac-*salt* overexpression via activating *dFOXO/PGC-1α* and blocking *TOR/*oxidative stress (Figure 6). LTE also improved age-related mobility decline and lifespan in cardiac *CG2196(salt)* overexpression or knockdown flies.

## MATERIALS AND METHODS

### *Drosophila* stock and diet

The *salt*-UAS-RNAi fly(Genotype:*w^1118^; P{GD12732}v28349/TM3;* stock ID: v28349) was obtained from the Vienna Drosophila RNAi Center. The *salt*-UAS-overexpression fly (Genotype: w^*^; TI{TI}mir-1014^KO^ salt^mir-1014-KO^; stock ID: 58888) was obtained from the Bloomington Stock Center. The *w^1118^* fly and *hand-Gal4* fly were gifts from Xiu-shan Wu (Heart Development Center of Hunan Normal University).

The *w^1118^* virgin female flies were divided into 3 groups: *w^1118^*, *w^1118^*+HSI(high-salt intake), and *w^1118^*+HSI+E(exercise). For the transgenic groups, maternal origin was selected as the genetic control group to avoid the influence of genetic background differences on the results. The “UAS-*salt*-overexpression virgin female flies”, “*hand-gal4>UAS-salt-overexpression* virgin female flies”, and “*hand-gal4>*U*AS-salt-overexpression* exercised virgin female flies” were respectively represented as “*salt*-control”, “*salt*-OE”, and “*salt*-OE+E”. Besides, The “UAS-*salt*-RNAi virgin female flies”, “*hand-gal4>*UAS-*salt*-RNAi virgin female flies”, “*hand-gal4>*UAS-*salt*-RNAi HSI virgin female flies”, and “*hand-gal4>*UAS-*salt*-RNAi HSI exercised virgin female flies” were respectively represented as “*salt*-control”, “*salt*-RNAi”, “*salt*-RNAi +HSI”, and “*salt*-RNAi+HSI+E”.

Normal food contained 2% agar, 10% sucrose, and10% yeast [54]. During the experiment, all experimental fruit flies were housed in a 12-h light/dark cycle and a 25° C incubator with 50% humidity. All experimental fruit flies were fed fresh food every other day during the experiment. High-salt foods were prepared by adding 2% of salt (NaCl) to normal foods [18]. All experimental flies would be provided with 1ml of pure water to drink, which was injected into a sponge plug. Compared with Stergiopoulos’study, our experimental conditions could be regarded as a mild high-salt diet since they did not provide pure water for *Drosophila* on the high-salt diet [12].

### Exercise training protocols

**Figure 7:**
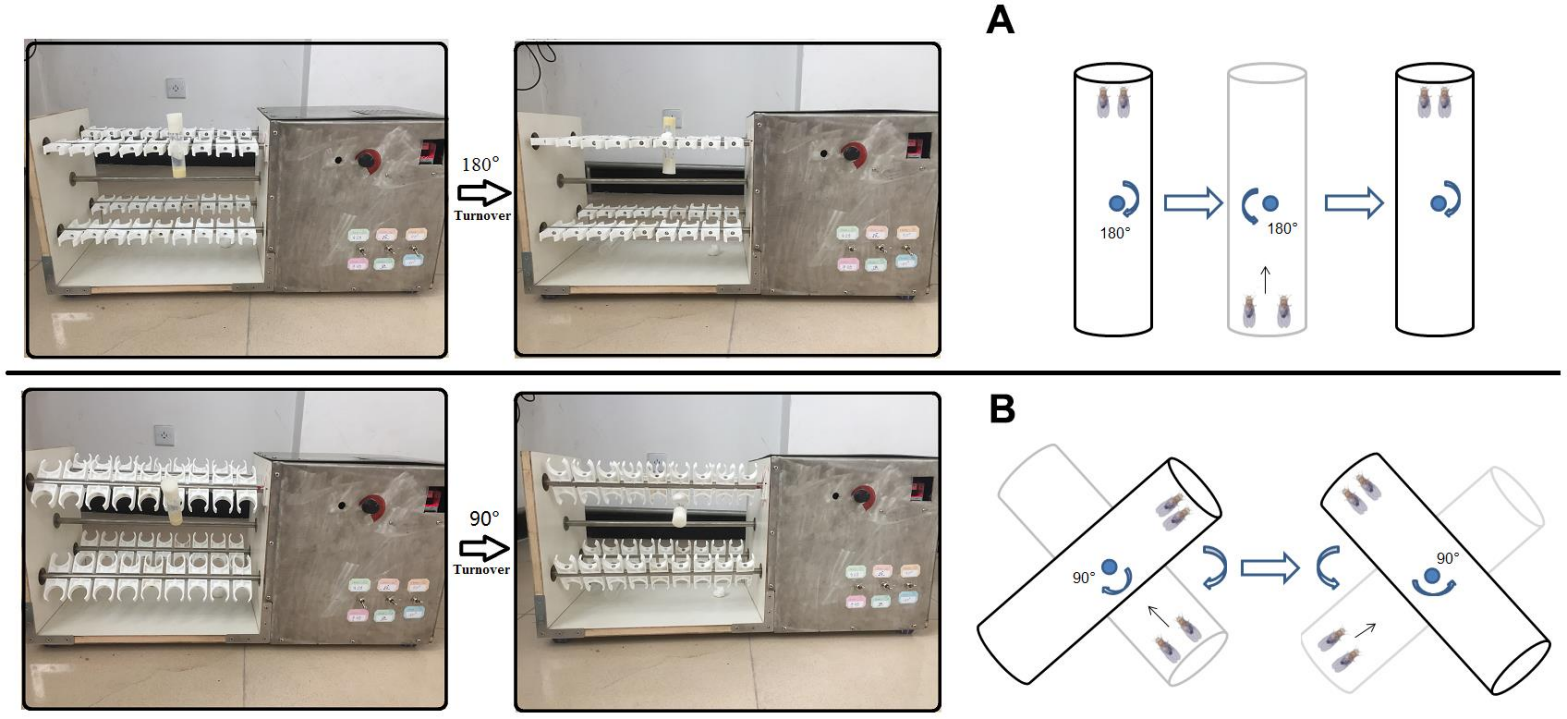
**Exercise training device.** (**A**) For young and adult flies, vials were vertically loaded in exercise device, and rotated 180° to make flies constantly climb (Just as Power Tower, overcoming weight = total body weight). (**B**) For aged flies (From 4-week to 5-week old flies in this study), vials were loaded in exercise device, and their long axis is at an Angle of 45° degrees to the horizontal plane (Overcoming weight = total body weight ×sin45°). When aged flies climbed and reached the top of vial, the vial were rotated 90° degrees to make flies constantly climb.

Using the characteristics of *Drosophila* negative geotactic, *Drosophila* in exercise group underwent climbing training for 5 weeks. The exercise group began to exercise at 1 week of age and ended at the end of 5 weeks of age. The flies in the exercise group were trained for 1.5 hours a day for five days a week and then rested for two days. The flies were trained to climb in a 10cm vials. After each reversal of the vials, the flies had 12 seconds to climb. The vials rotated at a rate of the 0.16 rev/s. *Drosophila* exercise training device was independently developed according to Power Tower and Tread Wheel principles (Figure 7) [65–67].

### Analysis of cardiac function

The head, ventral thorax, and ventral abdominal cuticle of anesthetized flies were removed, and the hearts were exposed. Oxygenated artificial hemolymph could maintain the normal function of the heart in *Drosophila.* High-speed cameras captured video of a fruit fly’s heart beating, and the video was shot at 120 FPS and took 30 seconds. The video of the heart beating was analyzed using SOHA software. The heart period, systolic period, diastolic period, fractional shortening, diastolic diameter, systolic diameter, and arrhythmia index could be measured by this method [68].

### The qRT-PCR assay

The flies’ hearts were placed in Trizol, and hearts were homogenized. The total RNA was purified from the Trizol by organic solvent extraction. The RNA was used to generate oligo dT-primed cDNAs after being treated with DNase I. In here, internal reference was Rp49 gene, and the quantity of total RNAs was normalized by Rp49. The SYBR green was used to measured Real-time PCR. Gene expression was calculated based on CT values. Primer sequences of *salt*: F: 5′-TTAATCGCAGGCGCGTCAGTG-3′; R: 5′-GGACGAGACCACCGTGTTAATCAG-3′. Primer sequences of *dFOXO*: F: 5’-AACAACAGCAGCATCAGCAG-3’; R: 5’-CTGAACCCGAGCATTCAGAT-3’. Primer sequences of *dTOR*: F: 5’- GAATTGTGGGCAGATGACCT-3’; R: 5’- CCTGCCTGTTGCA CTGATTA-3’. Primer sequences of *PGC-1*: F: 5’-TGTTGCTGCTACTGCTGCTT-3’; R: 5’-GCCTCTG CATCACCTACACA-3’. Primer sequences of *Rp49*: F: 5′-CTAAGCTGTCGCACAAATGG-3’; R: 5’- AACT TCTTGAATCCGGTGGG-3’.

### The SOD and MDA assay

The flies’ hearts were placed in PBS (pH 7.2–7.4), and these hearts were homogenized by freezing repeatedly with liquid nitrogen. The homogenate maintained at 2° C -8° C after melting. Next, the homogenate was centrifuged for 20 minutes at a speed of 2000-300rmp, and then the supernatant was removed. The methods and steps of SOD and MDA detection were strictly according to the operation requirements of the kits, and the insect SOD activity ELISA Kits and insect MDA ELISA Kits were provided by MLBIO(Shanghai, China) [18].

### Fatigue assay

The fatigue assay mainly based on the research of Tinkerhess MJ et al [65]. In a nutshell, 10 vials containing 20 flies each were were placed on the training device, and these flies were trained to be tired. When five flies or fewer than five flies were climbing in a vial, we considered the flies of this vial to be exercise-trained to fatigue, and we recorded the duration of exercise training as fatigue time of this vial. The training fatigue of *Drosophila* was observed and recorded every 30 minutes. The log-rank analysis was used to analyze and compare the differences in fatigue time.

### Climbing index assay

The climbing index assay mainly based on the research of Tinkerhess MJ et al [65]. In short, twenty fruit flies were placed in an 18-cm-long vial with an inner diameter of 2.8 cm. Before assessing negative geotaxis, flies were allowed to adapt to the vial for 10 minutes. A light box was placed behind the vials. Once the flies have been flipped back to the bottom of the vials, a picture was snapped by a timed digital camera after 8 seconds. 5 pictures were taken of each test vial. The climbing index was calculated based on the average climb height of the flies in the pictures.

### Longevity assays

The number of flies that died was recorded daily by the experimenters. The average lifespan of each group was calculated based on the survival time of each fruit fly. The survival curve of each group was made according to the survival time of each fruit fly. The log-rank analysis was used to analyze and compare the differences in longevity. Between 200 and 210 flies in each group were tested for longevity [69].

### Statistical analyses

Using a one-way analysis of variance (ANOVA) followed by an LSD test among different groups of the same age flies. The differences between 1-week-old flies and 5-week-old flies were verified by using an independent-sample *t* tests. P-values for lifespan curves and climbing endurance curves were calculated by the log-rank test. The GraphPad Prism and Social Sciences (SPSS) version 16.0 were used for statistical analysis. The statistical significance was set at *P<0.05*. Data are represented as means ± SEM.
